# Current Status of Rabies and Its Eradication in Eastern and Southeastern Europe

**DOI:** 10.3390/pathogens10060742

**Published:** 2021-06-12

**Authors:** Ivana Lojkić, Ivana Šimić, Tomislav Bedeković, Nina Krešić

**Affiliations:** 1 Laboratory for Rabies and General Virology, Department of Virology, Croatian Veterinary Institute, 10000 Zagreb, Croatia; bedekovic@veinst.hr (T.B.); lemo@veinst.hr (N.K.); 2 Laboratory for Molecular Virology, Division of Molecular Medicine, Ruđer Bošković Institute, 10000 Zagreb, Croatia; ivanasimzg@gmail.com

**Keywords:** sylvatic rabies, Croatia, Southeast Europe, oral rabies vaccination (ORV), bat rabies

## Abstract

The objective of this paper is to provide an overview of the current status of rabies in Europe, with special emphasis on Croatia and Southeast and East Europe. Due to the systematic implementation of a rabies eradication program by oral vaccination of wild animals, by the end of the 20th century, most West and Central European countries were rabies-free. The EU goal was to eradicate rabies in wildlife and domestic animals by 2020. No matter how achievable the goal seemed to be, the disease is still present in the eastern part of the EU, as was notified in 2020 by two member states—Poland and Romania. Croatia has been rabies-free for the last seven years but given that it borders a non-EU country in which a case of rabies was confirmed in 2020, it will continue to contribute to the maintenance of the rabies-free region. A rabies-free EU can only be achieved by continuous oral vaccination, coordination and a regional approach. The prevention of reintroductions from bordering countries in which rabies has not been eradicated yet, and the support for the eradication efforts made by these countries, are goals still pending.

## 1. Introduction

Rabies is a deadly zoonosis caused by a virus of the *Lyssavirus* genus belonging to the *Rhabdoviridae* family. Fortunately, the disease can be successfully prevented by appropriate pre- and post-exposure prophylaxis. However, less developed, and underdeveloped countries are still not able to implement such protection, meaning over 59,000 people die of rabies every year all over the world. Globally, urban rabies continues to be the greatest problem in underdeveloped countries, and the World Organization for Animal Health (OIE) has set 2030 as the deadline for its eradication [1]. The goal to eliminate sylvatic rabies from the European Union (EU) territory was set for 2020 [2]. Although the EU is on the verge of victory in the struggle against sylvatic rabies, further efforts still need to be made by some countries, particularly those close to the eastern EU borders. In this review, we will emphasize the importance of what has been achieved insofar but also to discuss potential dangers of rabies recurrence in Europe. 

## 2. Elimination of Sylvatic Rabies Using Oral Vaccination of Foxes

**Figure 1:**
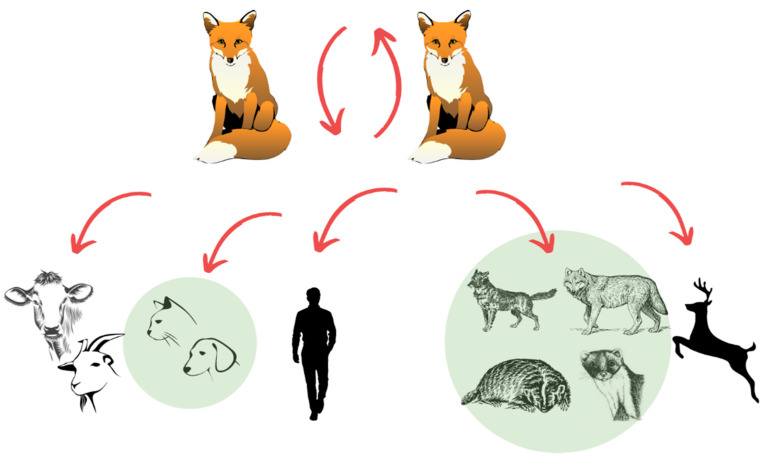
RABV transmission and perpetuation within the population of the primary carnivore reservoir host (red fox). From the primary reservoir, the virus is sporadically transmitted to other wild and domestic animals and to humans. Domestic and wild carnivores (green shaded circles) also sporadically transmit RABV to humans and other domestic animals.

Sylvatic rabies is caused by the classic rabies virus (Rabies virus, RABV). In Europe, the primary reservoir and vector of sylvatic rabies is the red fox (*Vulpes vulpes*) (Figure 1). The beginning of the spread of sylvatic rabies was recorded during World War II, when the first cases were discovered at the former Russian-Polish border [3]. The disease gradually and progressively spread from Northeast to Central and Southwest Europe [4,5]. The first country to launch a massive campaign for oral rabies vaccination (ORV) using attenuated vaccines [6] was Switzerland, back in 1978. In the EU countries, large-scale wildlife rabies eradication programs using ORV started in 1989 thanks to EU funding [7] and were soon proven to be the only efficient means of disease control [8]. 

ORV campaigns basically target red foxes and, to a lesser extent and following animal geographic expansions, raccoon dogs (*Nyctereutes procyonoides*) and golden jackals (*Canis aureus*). Oral vaccination baits normally contain a plastic sachet filled with live attenuated RV vaccine in liquid form, derived from the SAD-Bern strain, covered with a coating attractive to target species [9]. Bait casing usually contains 150 mg of tetracycline as a biomarker [7]. Aerial distribution of the vaccine baits is undertaken twice a year, in spring and autumn, in line with the red fox ecology [10,11] and optimal temperature conditions. Depending on the fox population density and the possible existence of persistent residual foci, densities of 20, 25 or even 30 baits per km^2^ are usually recommended [9,12]. To evaluate the efficacy of an ORV campaign, passive surveillance and active monitoring (bait uptake and immunization rate) are carried out. The methods to measure the antibody response after vaccination of target species, recommended by the OIE, are virus neutralization tests and ELISA [13]. Bait uptake is determined by screening for tetracycline in the teeth, the best long-term post-mortem tissue biomarker included in the vaccine bait. Given the above, the importance of vaccine titer checks before spreading (TCID50/bait), cold chain maintenance and timely control of bait distribution should not be neglected either. The results obtained by all these procedures show whether the ORV distribution methods were effective in achieving an adequate vaccination coverage and a decline in rabies incidence in the target population [14,15,16,17]. Nevertheless, the decline in rabies cases is an actual indicator of the efficacy of the ORV, and a prerequisite for this is a high and stable number of samples under surveillance and monitoring.

EU financing of eradication activities is of the utmost importance for the implementation of ORV programs. Since 2010, the EU has co-covered 75% of the costs of rabies eradication programs carried out in the member states. The EU also reimburses costs imposed by vaccination belt establishment along common borders with neighboring non-EU member states in Eastern Europe. At the beginning of the implementation of rabies eradication programs in Europe, the annual cost of such a program per country was between 10 and 16 million euros, depending mainly on the price of the bait, but also on land area and the length of the boundaries [18]. In Croatia, the ORV program started in the spring of 2011 and was co-financed by the European Union’s Instrument for Pre-Accession Assistance (IPA) [19], initially only in the north and east of the country and, from autumn 2012 onward, all over the country except for islands. At the same time, the implementation of ORV started in Bosnia and Herzegovina, Serbia, Montenegro, Macedonia, Kosovo [19] and Albania (in 2014) [7]. Until then, the total price of an ORV program per year decreased, mostly due to the lower bait price. The average yearly cost in Croatia (spring and autumn campaign) was around 2 million euros, and during the first four years of implementation, 75% of the costs were covered by the EU through the Instrument for Pre-Accession Assistances. After 2014, the costs were covered by the national budget with the financial support of the European Commission [20]. Consequently, such a comprehensive epidemiological approach to ORV implementation resulted in an annual decrease in the number of positive foxes. The effort and financial support of the EU had, and still has, the main role in the eradication of sylvatic rabies in Europe. For various reasons, mostly political, procurement problems, such as bidding and contract closure-related issues, may arise, hindering EU financial support release.

## 3. Current Status of Rabies, with Emphasis on Croatia, Southeast and East Europe

In the long time-period that elapsed from the first case of sylvatic rabies in Croatia in 1977 to the last case detected in spring 2014 [20], several high infection peaks were recorded. The first was in 1982, when rabid foxes crossed the Sava River and went south, spreading at the same time to the Istria peninsula and Gorski Kotar [21,22]. By 1990, rabies had spread all over Croatia except for the islands [23]. The maximum of 325 positive out of 695 examined foxes (46.8%) was recorded in 1993 [24]. At the beginning of the 21st century, the number of positive animals stabilized at about 20% (449/2240), but in 2008–2009, rose again to over 30% (994/3051) [25]. Given these devastating figures, it was clear that Croatia, together with Western Balkan countries, posed a threat to neighboring countries in which ORV had been conducted for many years, undermining their ability to achieve a rabies-free status. Although the ORV program started in Slovenia as early as 1995, the last case was recorded in 2013. Italy, which has been rabies-free since 1997, reimplemented the program following the discovery of a positive fox in 2008 at the border with Slovenia [26]. Greece, which has been rabies-free since 1987, had to reapply ORV in 2013 on the belt with Macedonia and Albania due to the re-emergence of positive cases in 2012 [27]. 

**Figure 2:**
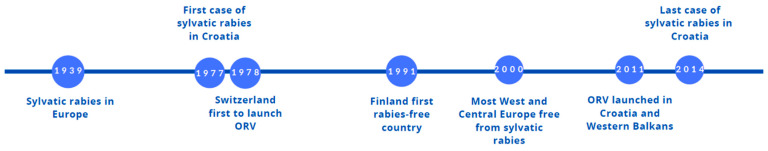
Timeline of the most important events related to the elimination of sylvatic rabies in European territory.

Over the past decade, the virus has been successfully eliminated from most West and Central European countries (Figure 2), and it is now restricted to the eastern part of the EU, where it was reported in 2019 and 2020 by only two member states—Poland and Romania. As for the non-EU member states, rabies cases were recorded in 2020 in Bosnia and Herzegovina, Georgia, Moldova and Ukraine [28]. In 2020, 5023 domestic and 21,910 wild animals (and 377 bats) originating from the European territory were tested. Only 12 endemic cases (six in wild animals and six in domestic animals) were detected, as follows: in five foxes, one dog and one cow in Poland, and in two dogs, two cows and one fox in Romania. Unfortunately, two rabies cases detected in Poland were identified in an area that had been rabies-free for more than 16 years prior to the event [29]. In 2020, one case was imported into France, most probably from Morocco [30]. The fact that no human cases were detected in 2020 (unlike 2019, when four human cases were imported into Italy, Latvia, Norway and Spain) [31] is very encouraging but might be the result of traveling restrictions imposed by the COVID-19 pandemic. 

**Figure 3:**
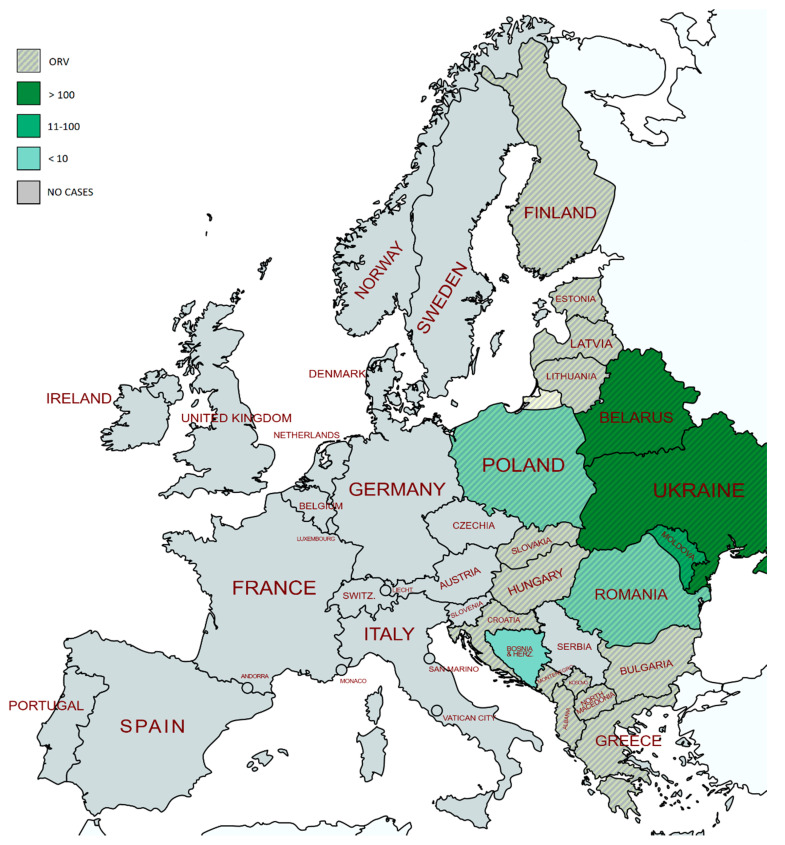
Geographical distribution of the reported rabies cases caused by RABV, and ORV programs running on European territory, in 2020.

In 2020, rabies national veterinary programs were continuously carried out in 11 member states and eight countries bordering the infected areas [32] (Figure 3). In 2021–2022, rabies eradication programs are planned for Albania, Bosnia and Herzegovina, Bulgaria, Croatia, Greece, Hungary, Montenegro, Poland, Romania, Serbia and Slovakia [33]. Missed campaigns had the most devastating impact on rabies eradication programs. On top of that, non-continuous monitoring and/or surveillance implying a decrease in the number of monitored samples can result in a failure to identify the remaining hotspots and hamper the freedom-from-rabies declaration. To substantiate this, we hereby give the most recent example of rabies re-emergence in Bosnia and Herzegovina. In July 2020 [34], a rabid dog was diagnosed only 30 kilometers from the location of the last recorded rabies case in Serbia in 2018 [35]. Unfortunately, due to economic reasons, the last vaccine baits in Bosnia and Herzegovina were distributed over its territory in spring 2018. Serbia, on the other hand, missed the 2017 autumn campaign and the entire 2020 campaign. Furthermore, there was no continuous monitoring/surveillance in Bosnia and Herzegovina. All of the above most likely enabled the persistence of a hotspot at the Serbia/ Bosnia and Herzegovina border. In 2021–2022, both Serbia and Bosnia and Herzegovina plan to implement the program [33], but the fact remains that Europe currently has a new rabies hotspot, without a continuous ORV program. Fortunately, this is not the case in Croatia, where the program shall be continued in 2021–2022. Should the program interruptions in Bosnia and Herzegovina go on, Croatia shall have to continue with the implementation of the rabies eradication program for many years to come. As recently stated by Černe et al. [36], although coordinated ORV campaigns started simultaneously in Slovenia and the neighboring countries Austria, Hungary and Italy back in 1988, ultimate success was achieved due to the participation of Croatia in the ORV program. The same can be applied to countries like Romania and Poland, which will win against sylvatic rabies only if Ukraine, Belarus and Moldova maintain ORV programs across their territories. In Romania, ORV was regularly implemented from 2014, and a gradual decline in the number of infected foxes was seen until 2018. In 2018, ORV was not implemented, and after that, the number of positive animals has stood at four to five per year. Still, all positive cases were close to the Moldavian and Ukrainian border. In the period of 2018–2020, Ukraine implemented only autumn campaigns with no campaigns implemented in two western regions [37]. The implementation of ORV in Moldova was planned for 2018–2020, but due to non-enforcement of the appropriate legislation, it started at the end of 2020 [38]. The above substantiates the fact that re-infections can only be prevented by continuous ORV implementation, coordination and a regional approach. 

## 4. Other Risks Should Not Be Neglected 

As discussed above, the main obstacles to maintaining freedom from rabies are non-continuous ORV program implementation, a decrease in the number of samples under monitoring, a lack of cooperation and of a regional approach. In this context, prevention of reintroductions from bordering countries in which eradication has not been achieved yet, and support for that eradication, are goals still pending. Despite considerable success in ORV implementation in Europe, the danger of reintroduction of the infection is always present. There are many recent examples of disease spread that occurred because of intentional movements of pets, livestock or wildlife. Population movements across national boundaries by wildlife vectors are important for the spread of the disease and have been studied extensively [3,39]. Both legal and illegal animal movements have resulted in the introduction of rabies to previously disease-free areas. One of the greatest risks of reintroduction of rabies into a rabies-free population is certainly the transport of unvaccinated animals from countries with endemic rabies. From 2006 to 2020, there were 19 such cases in Europe, of which as many as 16 occurred in dogs [32,40,41,42]. It happens mainly due to ignorance of the danger arising from the import of rabid or non-vaccinated animals, especially puppies, due to their attractiveness and small size. Such cases generally lead to secondary or even tertiary transmission of rabies, and consequently, to the loss of a country’s rabies-free status. The risk of infection of livestock should not be neglected either. This type of risk is closely related to the infection dynamics of disease reservoirs within a particular territory and the opportunities for contact between cattle and reservoir populations [43]. The only case in the European territory was reported in 2012 in Croatia after the import of livestock from Romania [44]. 

In view of possible threats, attention should be paid to other susceptible wildlife species, like badgers (*Meles meles*), martens (*Martes foina, Martes martes*), jackals (*Canis aureus moreoticus*), raccoon dogs (*Nyctereutes procyonoides*), small Indian mongoose (*Herpectes auropunctatus*) and last but most important, bats. On top of the major reservoir, i.e., red foxes, the most commonly infected wildlife species in many European countries are badgers (*Meles meles*) and martens (*Martes foina, Martes martes*) [24,45]. With the beginning of ORV implementation, the number of infected badgers and martens fell together with the number of infected foxes. Similar was seen with jackals (*Canis aureus moreoticus*), the species restricted to Southeastern Europe during the first half of the 20th century. Today, this species reaches even Estonia, the Netherlands and Denmark [46]. As their number in Croatia has increased during the last 15–20 years [45], jackal, along with red fox, have been recognized as a potential rabies reservoir species. Fox baits have also been shown to be attractive to jackals, as evidenced by the findings of biomarker tetracycline in the teeth of shot jackals when tested [47]. Unlike the rest of Europe, the raccoon dog (*Nyctereutes procyonoides*) is the main reservoir and carrier of rabies in Northeastern Europe and Poland. This invasive species progresses in its distribution by about 40 km, and up to 120 km, per year from the place of introduction, the former USSR [48]; nowadays, they are assumed to be distributed across the entire area between the Pannonian Plain and the Dinarides [49]. As these animals share their habitat with foxes, they have been shown to be successfully immunized using bait vaccines [50], hence decreasing the number of positives due to ORV [49]. Another invasive mammal species worth mentioning in this context is the small Indian mongoose (*Herpectes auropunctatus*), the most common carrier of rabies in the Caribbean [51]. This species was brought to the Croatian Island of Mljet as early as 1910 to reduce the number of snakes [52]. They have widely spread since and are currently present on southern Croatian islands and the coastal part of Dalmatia, Bosnia and Herzegovina and Montenegro [53]. As the Croatian islands have never seen rabies cases, mongoose populating them have been successfully used as a model to test the immunogenicity of baits used for oral vaccination of mongoose in the Caribbean [54,55]. Nevertheless, the species mentioned here do not necessarily represent a risk of re-emergence of rabies on the European territory, provided that the primary reservoir is successfully managed by ORV. 

However, ORV programs in Europe have proven useful in the control and eradication of rabies only in non-flying mammals. Flying mammals, bats, are the reservoir hosts of six currently identified species of bat lyssavirus in Europe [56] (*European bat lyssavirus type-1 (EBLV-1)*, *EBLV-2, Bokeloh bat lyssavirus (BBLV), West Caucasian bat lyssavirus (WCBV), Lleida bat lyssavirus (LLEBV)* and *Kotalahti bat lyssavirus (KBLV*)). Only EBLV-1, EBLV-2, BBLV and KBLV are classified in the same phylogenetic group (phylogroup I) of classical RABV, meaning that they can be effectively neutralized by currently available rabies vaccines intended for humans. EBLV-1, on several occasions, spilled over to domestic (cats, sheep) and wild animals (martens) and humans (two deaths), while EBLV-2 was reported in humans (two deaths) [57]. Active surveillance of rabies in bats through determination of anti-EBLV1 and EBLV2 antibodies is a powerful tool for the estimation of the number of bats that were in contact with the virus. A survey of a population of clinically healthy bats around Europe showed that seroprevalence depends on location, species, sex, social behavior (co-roosting), season (active season, hibernation period) and the intensity of rabies surveillance, and it varies from as low as 0.05% [58] to more than 60% [59]. In Croatia, a similar study showed the seroprevalence of specific antibodies in bats from various locations and habitats of 5.71 [60]. In light of the above, it is important to point out that bats in Europe will always pose a small but undeniable threat to human health [61]. This fact should not be neglected due to the existence of certain bat species that prefer to use human settlements as habitats, which allows their close contact with humans and domestic animals, especially cats. According to Rabies Bulletin Europe [28], 943 bats were tested in 2020, and 19 of them, originating from four European countries (Germany, Poland, the Netherlands and the United Kingdom) were virus-positive. The most recent case of rabies of bat origin was detected in a cat from Italy, in which the sequencing of the isolated virus showed 98.52% homology with the WCBL associated with Schreibers’ bent-winged bat (*Miniopterus schreibersii*) as the reservoir [62]. In view of the hunting nature of cats and the great migratory ability of bats, especially Schreibers’ bent-winged bat, it is easy to perceive the danger of transmission of not only rabies but also other viruses from bats to cats. 

## 5. Conclusions

Notwithstanding the great success of the EU ORV programs of sylvatic rabies eradication, the disease is still present in bats and will, therefore, never be completely eradicated in wildlife. Today, when almost the entire EU is free of the disease, the danger of reintroduction always emerges from countries with an unfavorable epidemiological situation, either via the transboundary route or human and animal trafficking. Oral vaccination of animals is the only effective tool to eradicate rabies, making its continuous implementation necessary. Rabies eradication also requires constant political and financial engagement of vulnerable countries, close cooperation between neighboring countries and political and financial EU support. Although no cases of rabies have been witnessed in Croatia since 2014, due to globalization and the possibility of disease spread by human and animal trafficking, it is important to continuously raise awareness among the profession and the public about the importance and the dangerous nature of this disease.

## Data Availability

Publicly available datasets were analyzed in this study. This data can be found here: http://www.who.int; https://ec.europa.eu; https://www.who-rabies-bulletin.org
